# Molecular Evolution of Slow and Quick Anion Channels (SLACs and QUACs/ALMTs)

**DOI:** 10.3389/fpls.2012.00263

**Published:** 2012-11-29

**Authors:** Ingo Dreyer, Judith Lucia Gomez-Porras, Diego Mauricio Riaño-Pachón, Rainer Hedrich, Dietmar Geiger

**Affiliations:** ^1^Plant Biophysics, Centro de Biotecnología y Genómica de Plantas, Universidad Politécnica de MadridMadrid, Spain; ^2^Molecular Biology of Winter Dormancy and Cold Acclimation in Woody Plants, Centro de Biotecnología y Genómica de Plantas, Universidad Politécnica de MadridMadrid, Spain; ^3^Grupo de Biología Computacional y Evolutiva, Departamento de Ciencias Biológicas, Universidad de los AndesBogotá DC, Colombia; ^4^Julius-von-Sachs Institute for Biosciences, Molecular Plant Physiology and Biophysics, Universität WürzburgWürzburg, Germany

**Keywords:** anion channel, evolution, SLAC/SLAH, ALMT, QUAC, voltage dependent, topology, phosphorylation

## Abstract

Electrophysiological analyses conducted about 25 years ago detected two types of anion channels in the plasma membrane of guard cells. One type of channel responds slowly to changes in membrane voltage while the other responds quickly. Consequently, they were named SLAC, for SLow Anion Channel, and QUAC, for QUick Anion Channel. Recently, genes *SLAC1* and *QUAC1/ALMT12*, underlying the two different anion current components, could be identified in the model plant *Arabidopsis thaliana*. Expression of the gene products in *Xenopus* oocytes confirmed the quick and slow current kinetics. In this study we provide an overview on our current knowledge on slow and quick anion channels in plants and analyze the molecular evolution of ALMT/QUAC-like and SLAC-like channels. We discovered fingerprints that allow screening databases for these channel types and were able to identify 192 (177 non-redundant) SLAC-like and 422 (402 non-redundant) ALMT/QUAC-like proteins in the fully sequenced genomes of 32 plant species. Phylogenetic analyses provided new insights into the molecular evolution of these channel types. We also combined sequence alignment and clustering with predictions of protein features, leading to the identification of known conserved phosphorylation sites in SLAC1-like channels along with potential sites that have not been yet experimentally confirmed. Using a similar strategy to analyze the hydropathicity of ALMT/QUAC-like channels, we propose a modified topology with additional transmembrane regions that integrates structure and function of these membrane proteins. Our results suggest that cross-referencing phylogenetic analyses with position-specific protein properties and functional data could be a very powerful tool for genome research approaches in general.

## Introduction

Patch-clamp studies with guard cells in the late 1980s and early 1990s showed that the guard cell plasma membranes harbor at least two types of anion channels (Schroeder and Hagiwara, 1989; Hedrich et al., 1990; Linder and Raschke, 1992; Schroeder and Keller, 1992). Based on the activation kinetics of the anion channel currents in response to voltage pulses, these were designated R(rapid) and S(slow)-type. Whereas the activation of R-type channels is in the low millisecond range, the transition of S-type channels to the open state takes several seconds (Linder and Raschke, 1992; Kolb et al., 1995). Furthermore, R-type channels display a pronounced voltage dependence. They are inactive at hyperpolarized membrane potentials but progressively activate upon depolarization (Hedrich and Marten, 1993; Kolb et al., 1995). In contrast, S-type channels are only weakly voltage dependent. The voltage dependent gating of both anion channel types is strongly modulated by the external anion activity (Hedrich and Marten, 1993; Hedrich et al., 1994; Lohse and Hedrich, 1995; Dietrich and Hedrich, 1998). Thus, the anion species and its concentration determine permeation and gating of these channels. It was shown, for instance, that malate represents not just a major substrate for R-type channels but also a gating modifier. This organic anion shifts the voltage dependent open probability of R-type channels to more negative voltages as it modifies the resting potential window of guard cells (Raschke, 2003). S-type currents are predominantly carried by chloride and nitrate ions instead (Schmidt and Schroeder, 1994). Similar to the situation with R-type channels, it has recently been shown that S-type channel gating is also modulated by permeating anions (Geiger et al., 2009, 2011).

**Figure 1:**
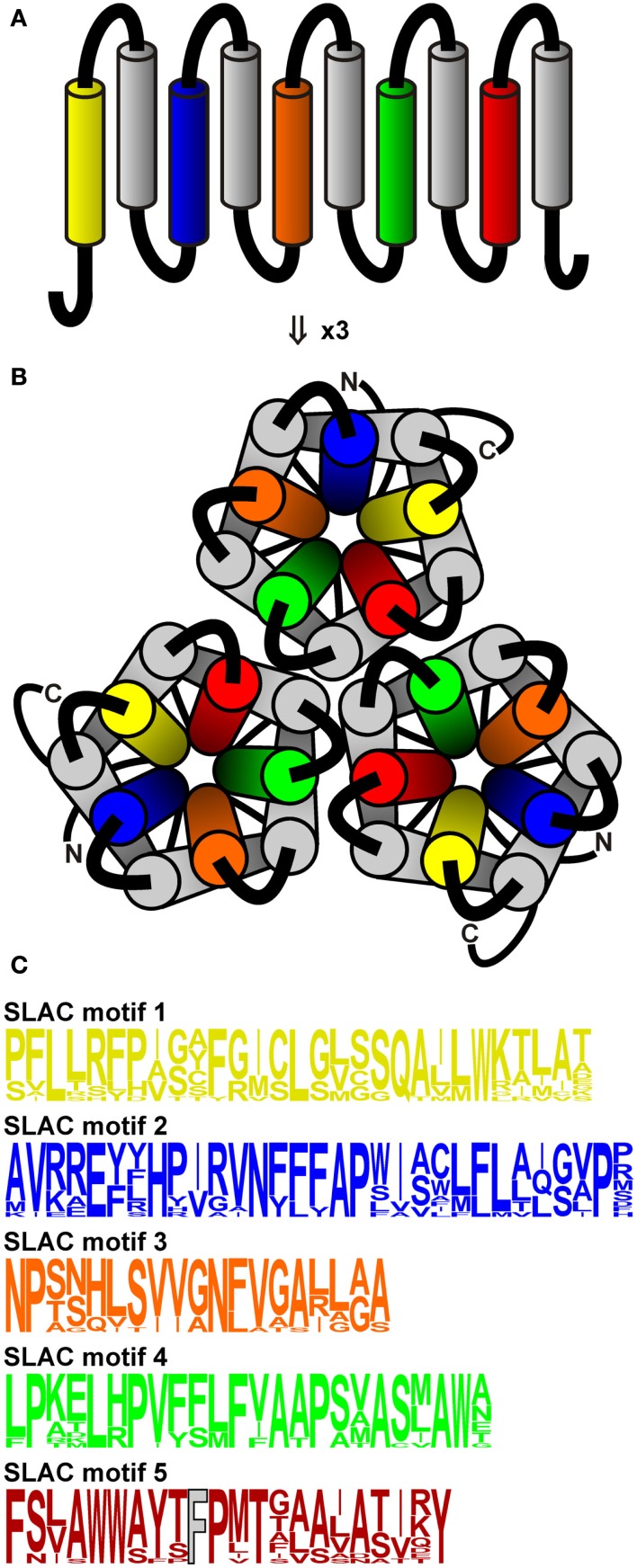
**Topology of SLAC-like channels. (A)** SLAC-like channels show a topology of ten transmembrane regions and cytosolic N- and C-termini. **(B)** Three channel modules assemble into a trimeric structure. The pore-forming transmembrane regions (yellow, blue, orange, green, red) were used to generate Hidden Markov Models (HMMs) to screen for SLAC-like channels in plant genomes. **(C)** Sequence logos of the HMMs of the regions shown in **(A)** and **(B)**. The crucial phenylalanine residue in the motif 5 (corresponding to Aratha-SLAC1-F450) is displayed in gray.

Following the discovery of anion channel currents in plants, it took almost two decades to identify the underlying genes encoding S and R-type anion channels. In 2008, the molecular nature of the guard cell slow anion channel from *A. thaliana* was uncovered using two independent screens: one for ozone-insensitive open-stomata mutants and the other for mutants impaired in CO_2_-dependent leaf temperature change (*oz* and *cdi3*, respectively; Negi et al., 2008; Saji et al., 2008; Vahisalu et al., 2008). When it became clear that the defect in the *cdi3/oz* gene affected distinctive properties of S-type channels in guard cells, the gene was (re)named *SLAC1* (slow anion channel) according to the nomenclature proposed by Klaus Raschke (Linder and Raschke, 1992). SLAC1 shares homology to the tellurite-resistance/C(4)-dicarboxylate transporters expressed in bacteria, archaea, and fungi. Recently, the crystal structure of one member of this family, HiTehA from *Haemophilus influenza*, was obtained (Chen et al., 2010). Using this structure as a template, a molecular model of SLAC1 was calculated. According to the structure of HiTehA, these channels are trimers composed of quasi-symmetrical subunits (Figure 1). Each subunit has 10 transmembrane helices arranged from helical hairpin pairs to form an inner five-helix transmembrane pore with a central conserved phenylalanine residue (*A. thaliana* SLAC1-F450) that could represent the anion gate. Indeed, mutation of the pore phenylalanine led to open anion conductance in both HiTehA and the flowering plant homolog SLAC1 (Chen et al., 2010). Besides the founding member SLAC1, that is exclusively expressed in guard cells, four SLAC1 Homologs were recognized in *A. thaliana* (SLAH1–4; Negi et al., 2008). Apart from SLAC1, SLAH3 is the only other S-type channel functionally characterized so far (Geiger et al., 2011); both channels differ significantly in their biophysical properties. In comparison to SLAC1, SLAH3 exhibits a higher preference for nitrate. In fact, for priming SLAH3 requires the presence of nitrate at its extracellular face. Nitrate thus functions as a substrate as well as a gate opener of SLAH3. SLAC1 and SLAH3 co-localize in the plasma membrane of guard cells, suggesting that upon stomatal closure they release chloride and nitrate in a concerted action (Negi et al., 2008; Vahisalu et al., 2008; Geiger et al., 2011). Lack of SLAC1 in Arabidopsis and rice was shown to result in stomata that appear not to close properly in response to high atmospheric CO_2_ levels, low relative humidity, and darkness (Negi et al., 2008; Vahisalu et al., 2008; Kusumi et al., 2012). Under the conditions tested so far, *Slah3* knockout plants did not show any stomatal phenotype. Patch-clamp studies, however, revealed that in *slac1* knockout plants S-type anion channels are active in nitrate-based buffers, whereas they are absent in guard cells of *slah3* loss-of-function plants (Geiger et al., 2011).

SLAC1 and SLAH3 both require interacting partners for opening. This may explain why initial attempts to express SLAC1 heterologously in *Xenopus* oocytes, for instance, did not result in the expected S-type anion conductance (Negi et al., 2008; Vahisalu et al., 2008). BiFC-based screens for interacting protein partners in oocytes followed by electrophysiological studies finally revealed the expected S-type activity. Both anion channels SLAC1 and SLAH3 interact with protein kinase-phosphatase pairs associated with abscisic acid (ABA) signaling (Geiger et al., 2009, 2010, 2011). In guard cells, and most likely all other plant cell types, increasing levels of the water stress hormone ABA as produced upon drought periods - is perceived in the cytosol (Levchenko et al., 2005) by members of the ABA receptor PYR/PYL/RCAR family (Ma et al., 2009; Park et al., 2009). Upon perception of ABA, the ABA receptor interacts with protein phosphatases of the PP2C family (ABI1) and suppresses their enzymatic activity. Phosphatase inhibition in turn enables activation of distinct SnRK- and/or CPK protein kinases. Among them, the SnR kinase OST1 and the CPK kinases 3, 6, 21, and 23 activated SLAC1, while SLAH3 was shown to be stimulated by CPK3, 6, 21, and 23 but not by OST1 (Geiger et al., 2009, 2010, 2011; Brandt et al., 2012; Scherzer et al., 2012). Following phosphorylation of the cytosolic N-terminal moieties of SLAC1 and SLAH3, the channels open very likely by a conformational change involving the phenylalanine gate. As a consequence, chloride and nitrate is released from guard cells and the membrane potential depolarizes. Depolarization activates Guard cell Outward Rectifying K^+^ channels (GORK, Ache et al., 2000; Hosy et al., 2003), leading to the release of potassium, thereby decreasing the osmotic potential and thus driving water out of the cell. The loss of guard cell turgor finally leads to stomatal closure.

Lately, ALMT12 from *A. thaliana* was identified as the guard cell quick anion channel or a prominent part of it. ALMT12 represents a member of the Aluminum activated malate transporters (ALMTs) family (Meyer et al., 2010; Sasaki et al., 2010). ALMT12 loss-of-function plants appeared impaired in malate-induced R-type anion currents in guard cells (Meyer et al., 2010) as well as in stimulus-induced stomatal closure (Meyer et al., 2010; Sasaki et al., 2010) indicating that the gene product of *ALMT12* is part of a channel complex that is involved in the release of anions from guard cells.

Al^3+^-activated malate transporters within this family were initially identified in wheat (*Triticum aestivum*; TaALMT1), rapeseed (*Brassica napus*), and *A. thaliana* (Sasaki et al., 2004; Hoekenga et al., 2006; Ligaba et al., 2006). ALMTs were shown to be involved in the detoxification of soil-borne Al^3+^, mediated by the release of malate that chelates the toxic cation (Ryan et al., 1997; Kollmeier et al., 2001; Ligaba et al., 2006; Delhaize et al., 2007). ALMT-expressing *Xenopus* oocytes were found to conduct Al^3+^-enhanced malate currents, in line with the physiological function of ALMT (Sasaki et al., 2004). Pre-incubation of oocytes with the protein kinase inhibitors K252 and staurosporine prevented Al^3+^-induced TaALMT1-derived currents (Ligaba et al., 2009), with Ser384 in the C-terminus of TaALMT1 identified as the key residue. In contrast to the well-known R-type features known from guard cells, the current response of TaALMT1 and homologs thereof did not show strong voltage sensitivity (Ryan et al., 1997; Kollmeier et al., 2001; Pineros and Kochian, 2001). In contrast, when expressed in *Xenopus* oocytes, ALMT12 reflected the hallmark electrical properties of R-type anion channels of Arabidopsis guard cell plasma membranes (Meyer et al., 2010). According to the nomenclature proposed by Klaus Raschke (Linder and Raschke, 1992), ALMT12 was renamed QUAC1 (QUick Anion Channel 1). This name better suits the function of the *ALMT12* gene product in guard cells, because QUAC1 is evidentially not activated by Al^3+^as the initially identified, eponymous ALMT family members, and moreover, QUAC1 operates as channel rather than transporter. Note, however, that both the QUAC1 channel and TaALMT1-type transporters share malate as a permeating ion.

Recently, two members of the Arabidopsis ALMT family have been shown to reside in the vacuolar membrane (Kovermann et al., 2007; Meyer et al., 2011). Patch-clamp experiments on vacuoles overexpressing ALMT6 revealed large channel-mediated inward-rectifying malate currents that appeared to be modulated by the vacuolar pH and the cytosolic malate concentration. Conversely, ALMT6 loss-of-function plants carry reduced malate currents (Meyer et al., 2011). ALMT9 shares its hallmark properties with ALMT6, but in contrast to the guard cell specific expression of ALMT6, ALMT9 was found to be expressed ubiquitously (Kovermann et al., 2007).

**Figure 2:**
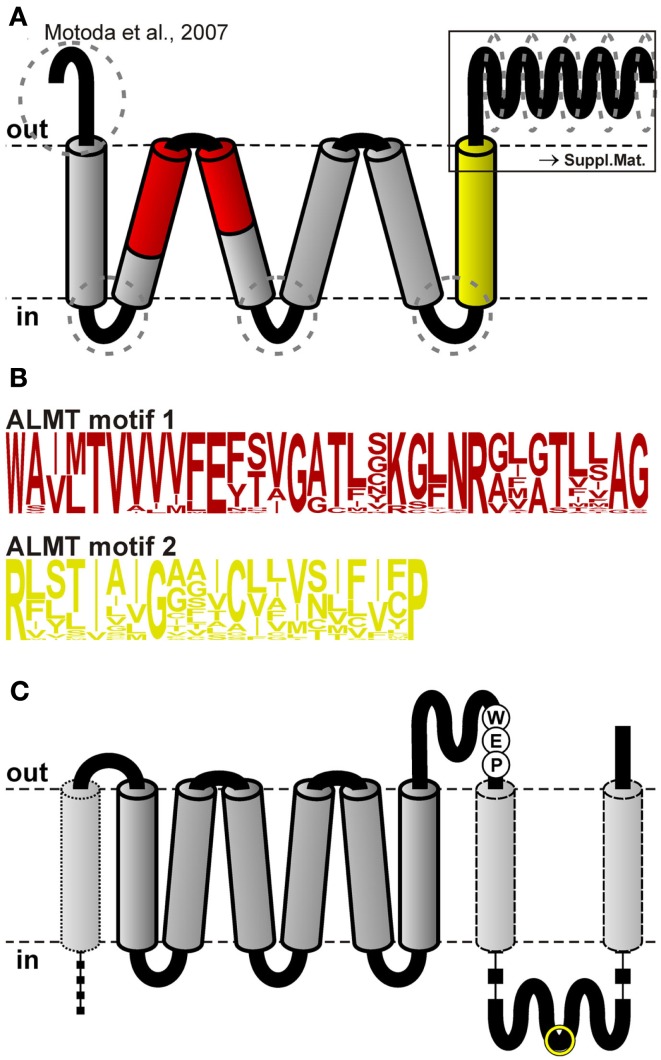
**Topology of ALMT/QUAC-like channels**. **(A)** Topology model developed by Motoda et al. (2007) for the channel TaALMT1. The red and the yellow regions were used to generate Hidden Markov Models (HMMs) to screen for ALMT/QUAC-like channels in plant genomes. Dotted ellipses indicate regions that differ most between the different clades of the ALMT/QUAC protein family. A detailed analysis of the C-terminal half is shown in Presentation S1 in Supplementary Material. **(B)** Sequence logos of the HMMs of the regions shown in **(A)**. **(C)** Proposed modified topology for ALMT/QUAC-like channels. In addition to the model of Motoda et al. (2007), the C-terminal half is spanning twice the membrane resulting in extracellular and intracellular C-terminal regions. Furthermore, the larger N-terminal extension may contain another membrane spanning region (dotted). The positions of the highly conserved WEP-motif as well as the position of the phosphorylation site TaALMT1-S384 (yellow circle) are indicated.

In contrast to the SLAC1 anion channel family, not even gross structural information for ALMT channels is available. From hydrophobicity considerations and subsequent immunocytochemical experiments, an initial topology model of TaALMT1 with six transmembrane helices and a long C-terminal domain was deduced (Figure 2A; Motoda et al., 2007). This model predicts the N- and C-termini to face the apoplast, which would put the key phosphorylation site Ser384 of TaALMT1 at the extracellular side, contrary to expectations, as it would not be accessible to protein kinases of the types known to control ion channel function in plants (Ligaba et al., 2009).

The comprehensive introduction presented so far summarized our current knowledge on slow and quick anion channels in plants in order to provide an overview also to readers who are not familiar with the field. In the following of this study we took the next step in gaining further detailed insights into the physiological role of SLAC and QUAC/ALMT proteins and their regulation. We investigated the molecular functional evolution of the two gene families by mining the available plant genomics data for valuable information on evolutionary conserved, potential functional domains in these proteins. The combination of phylogenetic analyses with predictions of protein features allowed to prognosticate putative phosphorylation sites in SLAC-like channels and to propose a new topology of QUAC/ALMT-like channels that reconciles the apparently contradicting experimental findings. Analyses of this type are intended to foster future structure-function studies on SLAC- and QUAC/ALMT-like channels, which, in the case of voltage-gated K^+^ channels, for instance, proved to be very successful for unveiling mechanisms of physiologically important regulations (Dreyer and Blatt, 2009; Dreyer and Uozumi, 2011).

## Results and Discussion

The protein classes of slow and quick anion channels show several characteristic sequence fingerprints. We used some of these sequence motifs (colored regions in Figures 1 and 2) and build Hidden Markov Models (HMMs) for these based on the known families of SLAC-like and ALMT/QUAC-like channels from a few model species. The HMMs (Figures 1C and 2B) were then employed to screen the deduced proteomes of two chlorophytes^1^, one bryophyte^2^, one lycophyte^3^, five Poaceae^4^, five Brassicaceae^5^, three Fabaceae^6^, two Euphorbiaceae^7^, two Rosaceae^8^, two Rutaceae^9^, one Salicaceae^10^, one Linaceae^11^, one Cucurbitaceae^12^, one Caricaceae^13^, one Myrtaceae^14^, one Vitaceae^15^, one Solanaceae^16^, one Scrophulariaceae^17^, and one Ranunculaceae^18^. In this process, duplicated database entries and a few false positives were removed from the data sets.

### The family of ALMT/QUAC-like proteins

In the model plant *A. thaliana*, the family of aluminum tolerance associated transporters (ALMTs) together with QUAC-like channels comprises 13 members (Hoekenga et al., 2006). Using BLAST with these as target sequences we identified 9 and 22 homologous channels/transporters in the genomes of *O. sativa* and *P. trichocarpa*, respectively. Based on this initial dataset we identified two regions as suitable for building HMMs for database searches. Motif 1 reaches from the middle of the second to the middle of the third transmembrane region (Figure 2, red) and motif 2 comprises the sixth predicted transmembrane region (Figure 2, yellow). Genome-wide screens spotted 400 non-redundant ALMT/QUAC-like proteins in 30 embryophyte species and two in two chlorophytes (Table S1 in Supplementary Material). Phylogenetic analyses assigned all of them to a single group of orthologs, indicating that the most recent common ancestor of all green plants comprised a single protein of the ALMT/QUAC type. Additionally, we realized that the basic structure of the phylogenetic tree can be inferred already by selecting ALMT/QUAC-like proteins of a few representative species. For better visualization of our conclusions we selected a set of 62 ALMT/QUAC-like proteins from *P. trichocarpa*, *P. patens*, and *S. moellendorffii* as well as *A. thaliana* (representing the Brassicaceae), *O. sativa* (for the Poaceae), and *M. truncatula* (for the Fabaceae).

**Figure 3:**
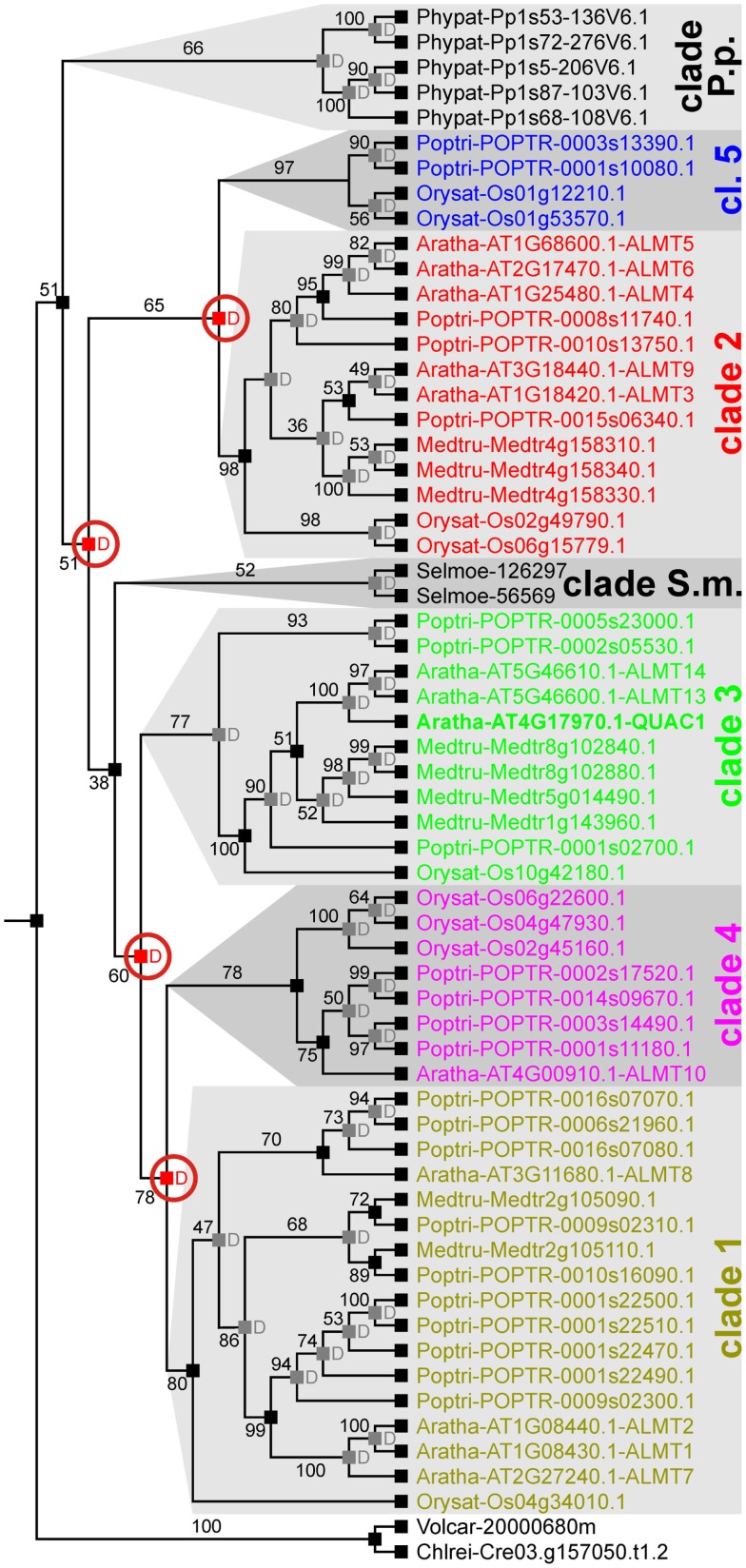
**Evolutionary relationships among ALMT/QUAC-like channels in land plants**. There are seven clearly distinguished clades of ALMT/QUAC-like channels in extant land plants, i.e., clades 1, 2, 3, 4, 5, clade S.m. (*S. moellendorffii* specific) and clade P.p. (*P. patens*-specific). Each clade represents an independent group of orthologs. To elaborate the evolutionary relationship, reconciliation analyses have been carried out. The last recent common ancestor of all embryophytes had a single ALMT/QUAC-like channel. Several duplications resulted in the diversity observed today in angiosperms. A *P. patens*-specific group separated relatively early. Right after, gene duplication separated clades 2/5 from the others. A second duplication separated clade 3 (comprising QUAC1/ALMT12) from clades 1/4 followed by a third duplication that then separated clade 1 from clade 4. In comparison quite recently, another duplication event led to the separation of clades 2 and 5. Red “D”s at branching points indicate predicted gene duplications, numbers designate bootstrap values. For clarity, predicted events of gene losses are not shown.

Previous phylogenetic analyses of ALMT/QUAC-like proteins from *A. thaliana*, *O. sativa*, and *P. trichocarpa* allowed subdividing this family into five clades (Barbier-Brygoo et al., 2011). Our analysis indicated that this classification is well suited to categorize these transporters from angiosperms. However, ALMT/QUAC-like channels from Bryophyta and Lycophyta cannot be grouped as such (Figure 3). Instead, proteins from *S. moellendorffii* and *P. patens* form separate groups of species-specific gene family amplifications.

Species tree vs. gene tree reconciliation analyses revealed that the functional and structural diversity of the class of ALMT/QUAC-like proteins derived from several gene duplication events in different lineages (Figure 3). An early duplication event before the emergence of lycophytes separated the groups 2/5 from 1/3/4, followed by the split of clade 3 by another duplication event after the emergence of lycophytes. Groups 1 and 4, and 2 and 5 divided thereafter. The separation of the groups is apparently combined by functional diversification. For instance, the proteins *A. thaliana* Aratha-ALMT6 and Aratha-ALMT9 (clade 2) are vacuolar malate-permeable channels (Kovermann et al., 2007; Meyer et al., 2011) whereas Aratha-ALMT1 (clade 1) and QUAC1 (=Aratha-ALMT12, clade 3) are located in the plasma membrane. Here, Aratha-ALMT1 mediates Al^3+^-induced malate extrusion (Hoekenga et al., 2006), while QUAC1 is an Al^3+^-insensitive voltage-gated anion channel (Meyer et al., 2010).

### Structural similarities and differences among QUAC/ALMT proteins

The available robust sequence information allowed spotting regions that might imply functional differences of the channels/transporters from the different clades in angiosperms. For analyses we used more than 300 putatively full-length QUAC/ALMT channel sequences of the five clades that did not show apparent deletions or potentially wrongly predicted splicing events.

**Figure 4:**
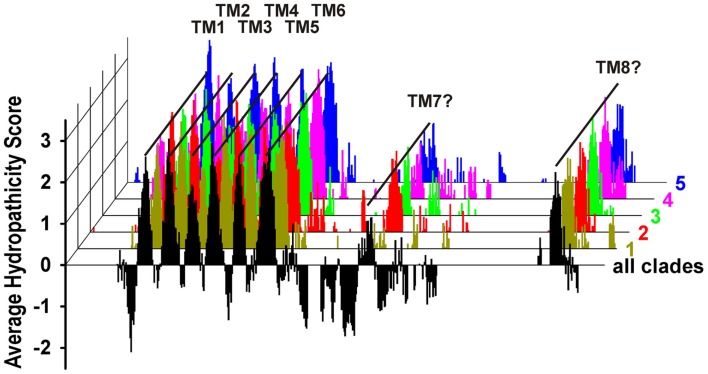
**Averaged Kyte and Doolittle plot of ALMT/QUAC-like channels**. The hydropathicity score was determined separately for each channel and then assigned to the respective position in the global sequence alignment. Subsequently, for each clade and for all channels the average values at each position were determined. The plot identified clearly the known six transmembrane domains TM1-TM6 and, additionally, two further potential transmembrane domains in the C-terminal halves of the proteins (TM7 and TM8). For clarity, negative values were only displayed for the average over all clades.

The sequence alignment indicated several highly conserved regions, such as the six predicted transmembrane domains and certain areas in the C-terminal extension. Most divergent regions were found at the N-terminus before the first transmembrane domain, in the cytosolic linkers between the transmembrane domains 1 and 2, 3 and 4, and 5 and 6, and within the C-terminal half of the proteins (Figure 2A, dotted circles). Especially, the latter region appears to be more structured than hitherto reported. Our alignment identified 10 separable zones of variable length with alternating higher and lower levels of conservation (29–50 vs. 17–28% identity; Presentation S1 in the Supplementary Material). Remarkable as fingerprints are the WEP-motif in the third zone, consisting of the highly conserved amino acid triplet Trp-Glu-Pro, and the conserved agglomeration of hydrophobic residues in the ninth zone. To evaluate whether the C-terminal halves of QUAC/ALMT proteins might contain overlooked transmembrane regions we combined our alignment with the hydropathicity scores of the single proteins. At each position average values were calculated for every clade and for all sequences. For further display only those positions were considered, where less than 25% of the sequences showed gaps. This analysis revealed that – besides the six known transmembrane domains in the first part of the proteins – two additional, conserved hydrophobic regions appeared in the C-terminal half (Figure 4): one is located right after the WEP-motif and the other coincides with the cluster of hydrophobic residues in the ninth zone. Based on these results we propose a modified topology for QUAC/ALMT channels, with the C-terminal half of the protein being partially extracellular and partially cytosolic (Figure 2C). This topology would be in line with all immunocytochemical results of Motoda et al. (2007) and would explain how the glutamate in the WEP-motif can be involved in external Al^3+^-activation of Aratha-ALMT1 and TaALMT1 (Furuichi et al., 2010) while TaALMT1-S384 (in the seventh zone) is a key residue for cytosolic phosphorylation processes (Ligaba et al., 2009). In the current model with the C-terminal half being exclusively extracellular these experimental findings are apparently contradictory. Future studies will have to provide further experimental evidence to distinguish between the different suggested topology models. They will also need to clarify whether the larger N-terminal extension is extracellular for all QUACs/ALMTs, as proposed for TaALMT1 (Motoda et al., 2007), or whether in this case there is also an additional transmembrane domain, at least in some channels/transporters (Figure 2C, dotted). In this context, it should be mentioned that so far no clear signal sequence for an extracellular localization of the N-terminal end of ALMT/QUAC channel proteins has been identified.

### The family of SLAC-like proteins

**Figure 5:**
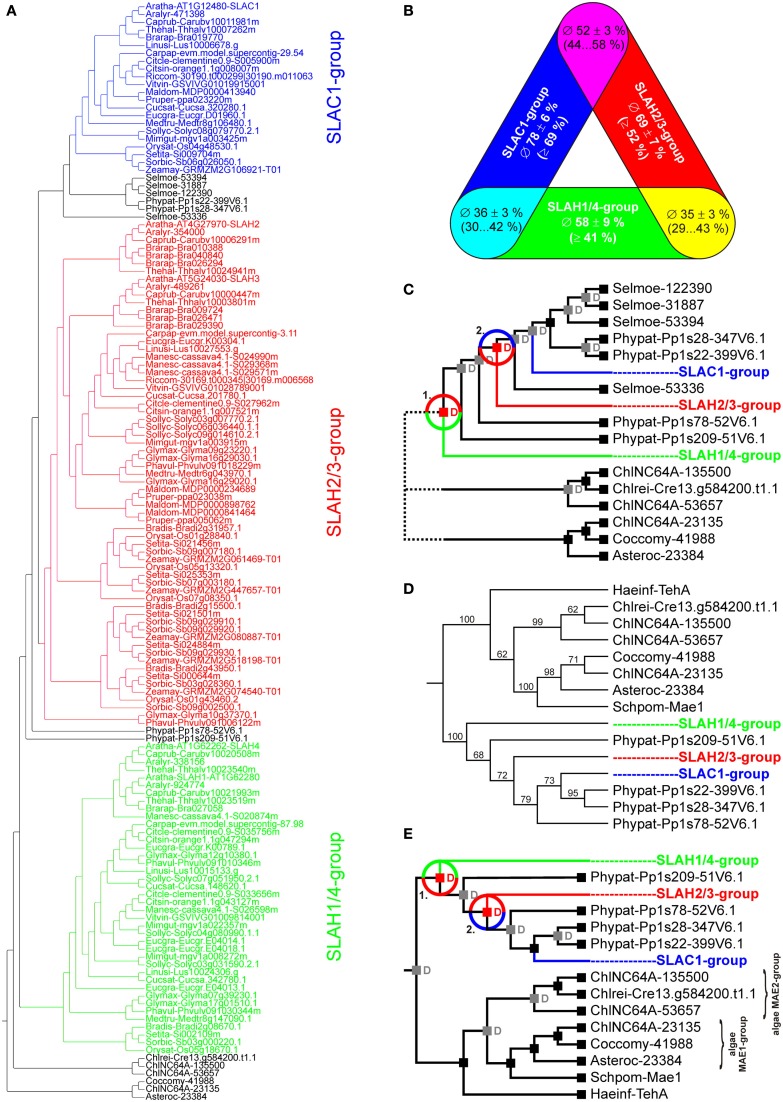
**Evolutionary relationships among SLAC-like channels in land plants**. **(A)** Phylogenetic tree resulting from UPGMA analyses of 135 SLAC-like channels that did not show apparent deletions or wrong predictions of splicing sites. SLAC-like channels of angiosperms could be grouped into three-groups: SLAC1-group (blue), SLAH2/3-group (red) and SLAH1/4-group (green). **(B)** Summary of pairwise identities of proteins within the three different groups and between the groups. Data are mean ± SD. In brackets the value range is specified. **(C)** Reconciliation analysis of early duplication events in SLAC-like proteins. SLAC-like channels diversified into the three groups by two duplication events (red “D”s and circles). For clarity, predicted events of gene losses are not shown. **(D)** Phylogenetic analysis of representative SLAC-like and algae-Mae-like channels. Aratha-SLAH1 represents the SLAH1/4-group, Aratha-SLAH2 the SLAH2/3-group and Aratha-SLAC1 the SLAC1-group. For comparison the tellurite-resistance/C(4)-dicarboxylate transporter Haeinf-TehA from *Haemophilus influenzae* and the malic acid transport protein Mae1 from *Schizosaccharomyces pombe* were included in the analysis. **(E)** Reconciliation analysis of the tree in **(D)** confirmed the results already obtained with the UPGMA data set. For clarity, predicted events of gene losses are not shown.

Earlier studies have identified SLAC1 homologs in Arabidopsis (*A. thaliana*), rice (*O. sativa*), grapevine (*V. vinifera*), and poplar (*P. trichocarpa*; Negi et al., 2008; Chen et al., 2010; Barbier-Brygoo et al., 2011). From these seed data sets we realized that SLAC-like proteins can be pinpointed by the presence of several conserved consensus motifs, which in turn might be exploited in genome-wide screenings. On the basis of the structural homology model of SLAC1 (Chen et al., 2010) we therefore generated Hidden Markov Models (HMMs) of the five transmembrane regions spanning the permeation pathway (Figure 1, colored). These HMMs allowed us to identify in total 176 non-redundant SLAC-like proteins in 29 species (Table S2 in Supplementary Material). However, this data set was not well suited for phylogenetic approaches because bootstrap analyses resulted – in part – in very low values, i.e., low statistical support of the tree structure. We therefore chose an alternative multi-step approach to fathom out the evolutionary relationship within this protein family. First, we identified the transmembrane segments and potential regulatory domains located in the cytosolic N-termini of the channels and extracted these regions for further analyses. For the channels of the model plant *A. thaliana* this corresponds to the amino acid stretches Aratha-SLAC1-K103_K516 (414 residues), Aratha-SLAH1-L26_K373 (348 residues), Aratha-SLAH2-D67_Q468 (402 residues), Aratha-SLAH3-D179_P583 (405 residues), and Aratha-SLAH4-L26_K365 (340 residues). Secondly, we determined by pairwise comparisons whether the respective sequences were complete. The gold standard in this context was provided by the five SLAC-like channels from *A. thaliana*. Potential deletions in the amino acid sequence might result from wrongly predicted splicing sites due to premature gene annotation, for instance. From the 176 SLAC-like channels, 135 appear to be complete in the selected sequence range. The sequences were then hierarchically clustered based on pairwise identities using UPGMA (Unweighted Pair Group Method with Arithmetic Mean). This clustering already provided us with a first picture of the phylogenetic structure of the SLAC-like channel protein family. Whereas channels from *S. moellendorffii* and *P. patens* take some intermediate position, channels of angiosperms could be clearly classified into three different similarity groups (Figure 5A): the SLAH1/4-group (green) contains Aratha-SLAH1 and Aratha-SLAH4; the SLAH2/3-group (red) with Aratha-SLAH2 and Aratha-SLAH3 and the SLAC1-group (blue) harboring Aratha-SLAC1, the funding member of the SLAC/SLAH family. Within these groups, channel identities are highly conserved ranging from ∼58% in the SLAH1/4-group, ∼69% in the SLAH2/3-group to ∼78% in the SLAC1-group (Figure 5B). In contrast, inter-group identities with ∼35% (SLAH1/4- vs. SLAH2/3-group), ∼36% (SLAH1/4- vs. SLAC1-group), and ∼52% (SLAH2/3- vs. SLAC1-group) are lower. Assuming that dispersion of identities is a measure for the age of a group, we may conclude the SLAC1-group to be the youngest and the SLAH1/4-group to be the oldest group of this protein family. This conclusion was further supported by reconciliation analysis of the data set (Figure 5C). The SLAH1/4-group apparently separated from the others already in a very early event before the appearances of bryophytes. From the available data it cannot be judged whether this process took place with the emergence of terrestrial plants or developed in the aquatic environment before colonizing the land. It is clear, however, that separation of the SLAH2/3-group from the SLAC1-group took place in a subsequent event on land after the appearance of bryophytes but before the occurrence of lycophytes. To further corroborate this hypothesis, we carried out detailed phylogenetic analyses with a condensed data set comprising SLAC-like channels from *P. patens* and one representative each from the SLAC1-group (Aratha-SLAC1), the SLAH2/3-group (Aratha-SLAH2), and the SLAH1/4-group (Aratha-SLAH1). Despite slight variations in the precise assignment of one channel from *P. patens*, the global tree structure and thus our picture on the evolutionary history of SLAC-like channels was confirmed (Figures 5D,E).

### Structural similarities and differences among SLAC-like proteins

**Figure 6:**
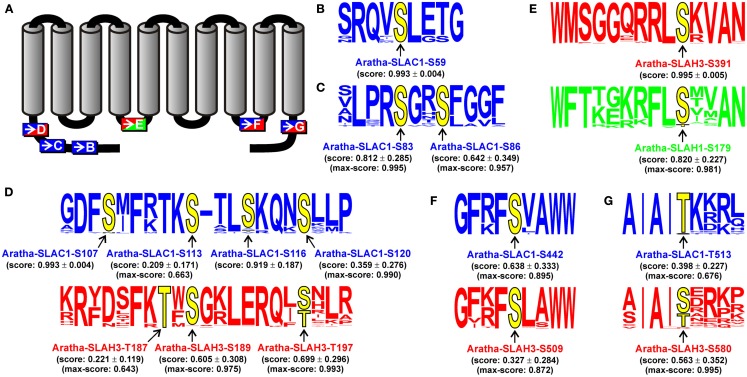
**Prediction of group-specifically conserved phosphorylation sites in SLAC-like channels**. The Netphos 2.0 score for S, T, and Y residues was determined separately for each channel and then assigned to the respective position in the global sequence alignment. Subsequently, the average values at each position were determined for each clade. Conserved positions were identified using the following criteria: (i) conservation of >90% of the potential phosphorylation site within one group, (ii) minimum score of 0.6 for at least one of these residues, and (iii) minimum average score of 0.2 within one channel group. **(A)** Illustration of the localization of the identified conserved phosphorylation sites. Letters refer to detailed information in **(B–G)**. **(B–G)** Sequence logos of the closer environment of the conserved putative phosphorylation sites (yellow). The positional information of the model channels Aratha-SLAC1, Aratha-SLAH3, and Aratha-SLAH1 is displayed explicitly. Values indicate mean ± SD of the score values. In case the mean score value was below 0.9, also the maximal value obtained for a single sequence is indicated. The displayed information is color coded (blue: SLAC1-group, red: SLAH2/3-group, green: SLAH1/4-group).

As in the case of QUAC/ALMT channels we also used the robust sequence information of SLAC-like channels to pinpoint regions and/or positions that might underlie functional variations between the groups. Most prominent differences between channels of the SLAH1/4-group and those of the other groups are (i) a shorter cytosolic C-terminus, (ii) a different initial stretch within the first transmembrane region TM1, and (iii) a much shorter cytosolic N-terminus. Especially the latter region was shown in Aratha-SLAC1 and Aratha-SLAH3 to contain important targets of kinases that regulate channel activity (Geiger et al., 2009, 2011; Vahisalu et al., 2010). This prompted us to exemplarily investigate the positional conservation of potential phosphorylation sites. We generated an alignment of the full-length sequences of the 135 SLAC-like channels mentioned above, combined this with the structure-based prediction of protein phosphorylation sites provided by Netphos 2.0, and obtained for each of the 12,155 Thr, Ser, or Tyr residues in the matrix an individual score. Further analyses were then limited to those residues that fulfilled all of the three following conditions: (i) >90% conservation of the potential phosphorylation site among all channels of one group, (ii) score >0.6 for at least one of these residues, and (iii) the average score >0.2 within a channel group. This screen for potential group-specific, conserved targets for kinases did not identify any tyrosine residue, but nine serine/threonine residues in channels of the SLAC1-group, six in the SLAH2/3-group and one in the SLAH1/4-group (Figure 6). Three SLAC1-group-specific potential phosphoserine residues were located in the first part of the cytosolic N-terminal region (S59, S83, and S86 in the model channel Aratha-SLAC1; Figures 6B,C). Interestingly, both regions of Aratha-SLAC1 have already been shown experimentally to be phosphorylated by the protein kinase OST1 (Geiger et al., 2009; S59 and S86, Vahisalu et al., 2010) and CPK6 (S59, Brandt et al., 2012). Another cytosolic region, short before the first transmembrane domain, contains several predicted, highly conserved phosphorylation sites in the two SLAC1- and SLAH2/3-groups (in Aratha-SLAC1: S107, S113, S116, and S120; in Aratha-SLAH3: T187, S189, T197; Figure 6D). Also, these regions had been experimentally proven to be phosphorylated by OST1 (Aratha-SLAC1, S120) and CPK21 (Aratha-SLAH3, T187), respectively (Geiger et al., 2009, 2011; Vahisalu et al., 2010). Besides these N-terminal regions, our screen also predicted a highly conserved phosphorylation site in the cytosolic C-terminus right after the last transmembrane domain of SLAC1- and SLAH2/3-like channels (Aratha-SLAC1-T513 and Aratha-SLAH3-S580; Figure 6G). In Aratha-SLAC1, this region was already identified as target of the kinase OST1 (Geiger et al., 2009). Thus, our unbiased prediction of group-specifically conserved phosphorylation sites matched perfectly the experimental findings obtained so far on the model channels Aratha-SLAC1 and Aratha-SLAH3. In addition, the screen predicts two further sites in the cytosolic linkers between the fourth and fifth and between the eighth and ninth transmembrane domains of SLAH1/4- and SLAH2/3-like (Figure 6E) and SLAC1- and SLAH2/3-like channels (Figure 6F), respectively. These data provide a valuable source of information for future studies that will focus *in vitro*, in oocytes, and *in planta* on single as well multiple phoshorylation site mutants in order to understand role and mechanism of the different SLAC/SLAH kinases.

### Evolutionary origin of SLACs and ALMTs/QUACs

Our analysis indicated that ALMT/QUAC-like channels have diversified from a single gene in the most recent common ancestor of green plants. The fact that one ALMT/QUAC ortholog each was identified in the chlorophytes *C. reinhardtii* and *V. carteri* adds substance to such an explanation (Figure 3, Table S1 in Supplementary Material). The analysis of homologs in higher plants is consistent with processes that involved several successive gene duplications since the colonization of the land environment. Interestingly, clades 2/5 split from the other three with or before the appearance of vascular plants. Clade 2 contains Aratha-ALMT6 and Aratha-ALMT9, both of which have been previously characterized at the functional level. Both ALMTs were localized in the tonoplast and typified as vacuolar malate channels rather than transporters (Kovermann et al., 2007; Meyer et al., 2011). Following clade 2/5, clade 3 (containing QUAC1) separated from clades 1 (containing ALMT1) and 4. Since ALMT1 is activated by Al^3+^ but QUAC1 is not, it remains an open question whether Al^3+^-activation in channels of the ALMT/QUAC-like protein family was lost at some point or developed later during evolutionary specialization.

**Figure 7:**
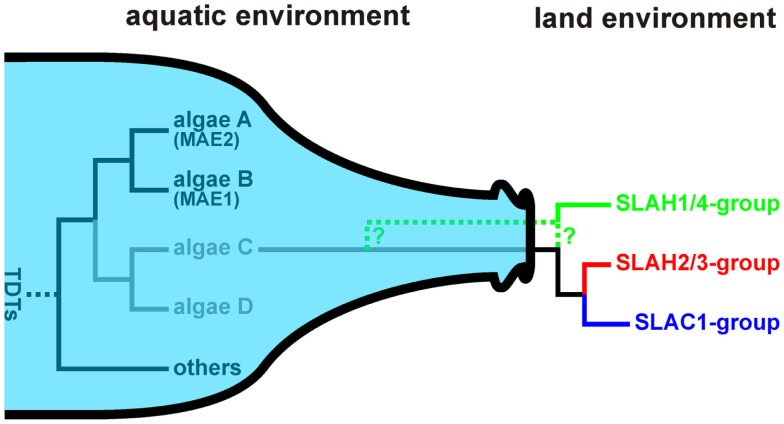
**Bottleneck model for the evolution of SLAC-like channels**. In the aquatic environment Tellurite-resistance/Dicarboxylate Transporters (TDTs) evolved into a huge variability. In algae transporters were identified that share features with malic acid transport proteins (MAEs). Closer relatives of the common ancestor of SLAC-like channels still need to be identified in Chlorophyta (algae C, algae D). Only one of these protein classes survived the transition from the aquatic to the land environment setting the starting point for the further evolution of SLAC-like channels.

The evolutionary path of SLAC-like channels is not as straight as it appears for the ALMT/QUAC-like proteins. In algae we identified two distinct groups of the Tellurite-resistance/Dicarboxylate Transporter (TDT) family (Figures 5A,D,E; Table S2 in Supplementary Material). None of those is a clade of SLAC-like orthologs but show closer similarity to the malate permease Mae1 from *Schizosaccharomyces pombe*. In some species, e.g., *Coccomyxasubellipsoidea* C-169, *Chlorella variabilis*, and *Asterochloris* sp., we identified only proteins of one group, in other species, e.g., *C. reinhardtii*, only members of the other. Interestingly, *Chlorella* NC64A comprises both groups in parallel, indicating that algae may exhibit a larger variability of TDTs. Thus, SLAC-like channels might have originated from another clade of this protein family still waiting to be identified in Chlorophyta. The diversity of TDT proteins in algae very likely collapsed with the transition of plants from an aqueous environment to one where water was often limiting. Functional diversification of SLAC-like channels in higher plants appears to have originated from only one group that survived the land transition (Figure 7). A similar bottleneck scenario was recently proposed for voltage-gated K^+^ channels (Gomez-Porras et al., 2012). This concept was challenged by screening databases of embryophytes for the presence of *S. pombe* Mae1-like proteins. As a matter of fact, we identified ESTs coding for such proteins also from higher plants, e.g., from clementine (*C. clementine*), apple (*M. domestica*), Japanese cedar (*Cryptomeria japonica*), the perennial grass *Cleistogenes songorica*, and common hop (*Humulus lupulus*). Closer inspection, however, revealed that the encoded proteins show best hits with very high sequence identities (50–90%) to annotated C4-dicarboxylate transporter/malic acid transporters from plant pathogens. Therefore we conclude that the few found ESTs originate from contaminations rather than from the host species themselves. Thus apart from SLAC-like channels, unequivocal evidence for the existence of other classes of the Tellurite-resistance/Dicarboxylate Transporter (TDT) family in embryophytes is apparently lacking.

## Summary

Slow and quick anion channels are essential for proper guard cell function (Kollist et al., 2011; Hedrich, 2012; Roelfsema et al., 2012). Both SLAC- and ALMT/QUAC-like proteins belong to larger protein families and each evolved from a common ancestor. Despite these parallels it is evident that SLAC-like channels show a higher degree of conservation than ALMTs/QUACs. Whereas SLAC-like proteins diversified via two duplication events, ALMT/QUAC-like proteins expanded by a rather early split followed closely by two subsequent events and a more recent final one.

Based on these phylogenetic analyses we hypothesize that group-specific evolution and conservation of structural and regulatory sites laid the ground for the development of unique properties associated with each channel type. The strategy of combining sequence alignment and clustering with predictions of local protein properties allowed us pinpointing potential phosphorylation sites in SLAC1-like channels in an unbiased manner. Several residues have been verified by site-directed approaches before. Experimental analysis of the remaining population of likely phosphorylation sites will help further understand multi-kinase activation of SLAC/SLAH channels (Scherzer et al., 2012). Using a similar strategy as with SLAC/SLAHs, we generated a modified topology model for ALMT/QUAC-like channels, which could serve as a valuable source for comparative physiological approaches and structure-function studies.

We are optimistic that cross-referencing phylogenetic analyses with position-specific protein properties together with subsequent experimental functional testing *in vitro*, in oocytes and finally *in planta*, will foster genome research approaches on plant ion channels and very likely on transporters and pumps as well.

## Materials and Methods

### Genome-wide search for SLAC- and ALMT/QUAC-like proteins

Putative ALMT/QUACs and SLACs were identified using the conceptual proteomes of *A. caerulea*, *A. lyrata*, *A. thaliana*, *B. distachyon*, *B. rapa*, *C. rubella*, *C. papaya*, *C. reinhardtii*, *C. clementina*, *C. sinensis*, *C. sativus*, *E. grandis*, *G. max*, *L. usitatissimum*, *M. domestica*, *M. esculenta*, *M. truncatula*, *M. guttatus*, *O. sativa*, *P. vulgaris*, *P. patens*, *P. trichocarpa*, *P. persica*, *R. communis*, *S. moellendorffii*, *S. italica*, *S. bicolor*, *T. halophila*, *V. vinifera*, *V. carteri*, and *Z. mays* (Phytozome v8.0), and the proteome of *S. lycopersicum* (SOL Genomics). Initially, we identified a starting data set obtained by BLAST searches in the genomes using known channels from *A. thaliana* as templates. Then, for each of the transmembrane domains 1, 3, 5, 7, and 9 of SLAC-like channels (Figure 1, colored) and of the two selected regions of ALMT/QUAC-like proteins (Figure 2, colored) a multiple alignment was created and an HMM built using the HMMER^19^ v3.0 package suite (Figures 1C and 2B). Thereafter, the HMMs were used to screen the deduced proteomes of the species under study. The criterion for inclusion was that at least one of the motifs must be present in the protein with a score >0.001. The entire protein sequence was then extracted. Retrieved proteins were curated in a semi-automatic way in order to eliminate false positives: the *n* protein sequences of each channel type of each species were pairwise aligned using Clustall W2^20^. From the resulting *n*(*n*-1)/2 pairs those with a score of <20 and of >97 (identical sequences) were removed. The residual pairs fragmented the sequences into distinct groups. That group with the highest similarity to the corresponding *Arabidopsis* channels was selected for further analyses. Proteins of the TDT family from algae were identified by blastp searches in the proteomes of *Asterochloris* sp. *Cgr/DA1pho*^21^, *Chlorella variabilis* NC64A^22^, and *Coccomyxasubellipsoidea* C-169^23^ using the malic acid transport protein Mae1 from *Schizosaccharomyces pombe* (NP_594777.1) as template. The NCBI accession number of Haeinf-TehA is NP_438669.

### Phylogenetic analyses

Sequences were aligned using the auto setting in MAFFT^24^ (Multiple Alignment with Fast Fourier Transform; Katoh and Toh, 2010) and the resulting alignments were analyzed by GBlocks (Talavera and Castresana, 2007), in order to keep robust regions for the phylogenetic inference. For the analysis presented in Figure 3, these were localized in the predicted transmembrane areas and in the zones 1 and 3 in the C-terminal part of the proteins (Presentation S1 in Supplementary Material). For the analysis presented in Figure 5D, these regions were localized in the transmembrane areas of SLAC-like and MAE-like channels. Sequence alignments are provided in DataSheet S1.FASTA and DataSheet S2.FASTA in the Supplementary Material. Evolutionary relationships were inferred by Maximum Likelihood using RAxML and 1000 bootstrap replicates (Stamatakis, 2006). The evolutionary model used for phylogenetic analyses was inferred using ProtTest (Darriba et al., 2011). In both cases, ALMTs/QUACs and SLACs, the evolutionary model determined by ProtTest was JTT + γ. In order to root and resolve the gene trees, we performed a gene tree-species tree reconciliation analysis using the species tree from from Phytozome v8.0^25^ and the Tree Of Life^26^. Reconciliation analysis was carried out in Notung 2.6 (Chen et al., 2000; Vernot et al., 2007). Events of gene losses were not shown. UPGMA (Unweighted Pair Group Method with Arithmetic Mean) analyses were carried out in MAFFT.

### Analyses of protein features

Protein hydropathicity at the amino acid scale were analyzed for each protein sequence separately with the web-based tool ProtScale^27^ using the “Kyte and Doolittle (1982)” option. The obtained values were then integrated into the global sequence alignment. If at a specific position of the alignment less than 25% of the sequences showed gaps, average hydropathicity scores were calculated. Results were qualitatively not different when – instead of 25% – any threshold between 1 and 95% was chosen.

The probability of an S/T or Y residue in SLAC1-like channels to be a protein kinase target was estimated with the web-based tool Netphos 2.0^28^. The obtained values were then integrated into the global sequence alignment and analyzed for group-specifically conserved phosphorylation sites as outlined in the text. Sequence logos of identified sites were generated with the web-based tool WebLogo^29^.

## Conflict of Interest Statement

The authors declare that the research was conducted in the absence of any commercial or financial relationships that could be construed as a potential conflict of interest.

## Supplementary Material

The Supplementary Material for this article can be found online at http://www.frontiersin.org/Plant_Traffic_and_Transport/10.3389/fpls.2012.00263/abstract

Supplementary Presentation S1 **Alignment of the C-terminal regions of ALMT-like proteins**. Based on the grade of conservation, the C-terminal regions of ALMT-like proteins can be divided into 10 zones. Zone 3 contains the highly conserved fingerprint amino acid triplet Trp-Glu-Pro (WEP-motif) and zone 7 contains the residue TaALMT1-S384 that was shown to be involved in phosphoregulation of this transporter. The two additional putative transmembrane regions that were identified in this study are indicated (TM7 and TM8).

Supplementary DataSheet S1.FASTA **Alignment of the sequence blocks used to generate the phylogenetic analysis shown in Figure 3**.

Supplementary DataSheet S2.FASTA **Alignment of the sequence blocks used to generate the phylogenetic analysis shown in Figure 5D**.

Supplementary DataSheet S3.XLSX **Supplementary Table S1 ALMT/QUAC-like channels presented in this study**. Channels were categorized according to Figure 3.
**Supplementary Table S2 SLAC-like channels presented in this study**. Channels were categorized according to Figure 5.

## Footnotes

^1^*Chlamydomonas reinhardtii* and *Volvox carteri*

^2^*Physcomitrella patens*

^3^*Selaginella moellendorffii*

^4^*Sorghum bicolor*, *Zea mays*, *Setaria italica*, *Oryza sativa*, and *Brachypodium distachyon*

^5^*Arabidopsis thaliana*, *Arabidopsis lyrata*, *Capsella rubella*, *Brassica rapa*, and *Thellungiella halophila*

^6^*Medicago truncatula*, *Phaseolus vulgaris*, and *Glycine max*

^7^*Manihot esculenta* and *Ricinus communis*

^8^*Prunus persica* and *Malus domestica*

^9^*Citrus sinensis* and *Citrus clementina*

^10^*Populus trichocarpa*

^11^*Linum usitatissimum*

^12^*Cucumis sativus*

^13^*Carica papaya*

^14^*Eucalyptus grandis*

^15^*Vitis vinifera*

^16^*Solanum lycopersicum*

^17^*Mimulus guttatus*

^18^*Aquilegia caerulea*

^19^http://hmmer.org

^20^http://www.ebi.ac.uk/Tools/services/web/toolform.ebi?tool=clustalw2

^21^http://genome.jgi-psf.org/Astpho1/Astpho1.home.html

^22^http://genome.jgi-psf.org/ChlNC64A_1/ChlNC64A_1.home.html

^23^http://genome.jgi.doe.gov/Coc_C169_1/Coc_C169_1.home.html

^24^http://mafft.cbrc.jp/alignment/software/

^25^http://www.phytozome.net/

^26^http://tolweb.org/tree/

^27^http://web.expasy.org/protscale/

^28^http://www.cbs.dtu.dk/services/NetPhos/

^29^http://weblogo.berkeley.edu/logo.cgi
