# Supplementary material for: Molecular Evolution of Slow and Quick Anion Channels (SLACs and QUACs/ALMTs)
Source: Front Plant Sci. 2012 Nov 29;3:263. doi: 10.3389/fpls.2012.00263 (PMC3509319; doi:10.3389/fpls.2012.00263)
Supplement: Supplementary Presentation S1 — Alignment of the C-terminal regions of ALMT-like proteins. Based on the grade of conservation, the C-terminal regions of ALMT-like proteins can be divided into 10 zones. Zone 3 contains the highly conserved fingerprint amino acid triplet Trp-Glu-Pro (WEP-motif) and zone 7 contains the residue TaALMT1-S384 that was shown to be involved in phosphoregulation of this transporter. The two additional putative transmembrane regions that were identified in this study are indicated (TM7 and TM8). [file 31606_Dreyer_Presentation1.PDF]

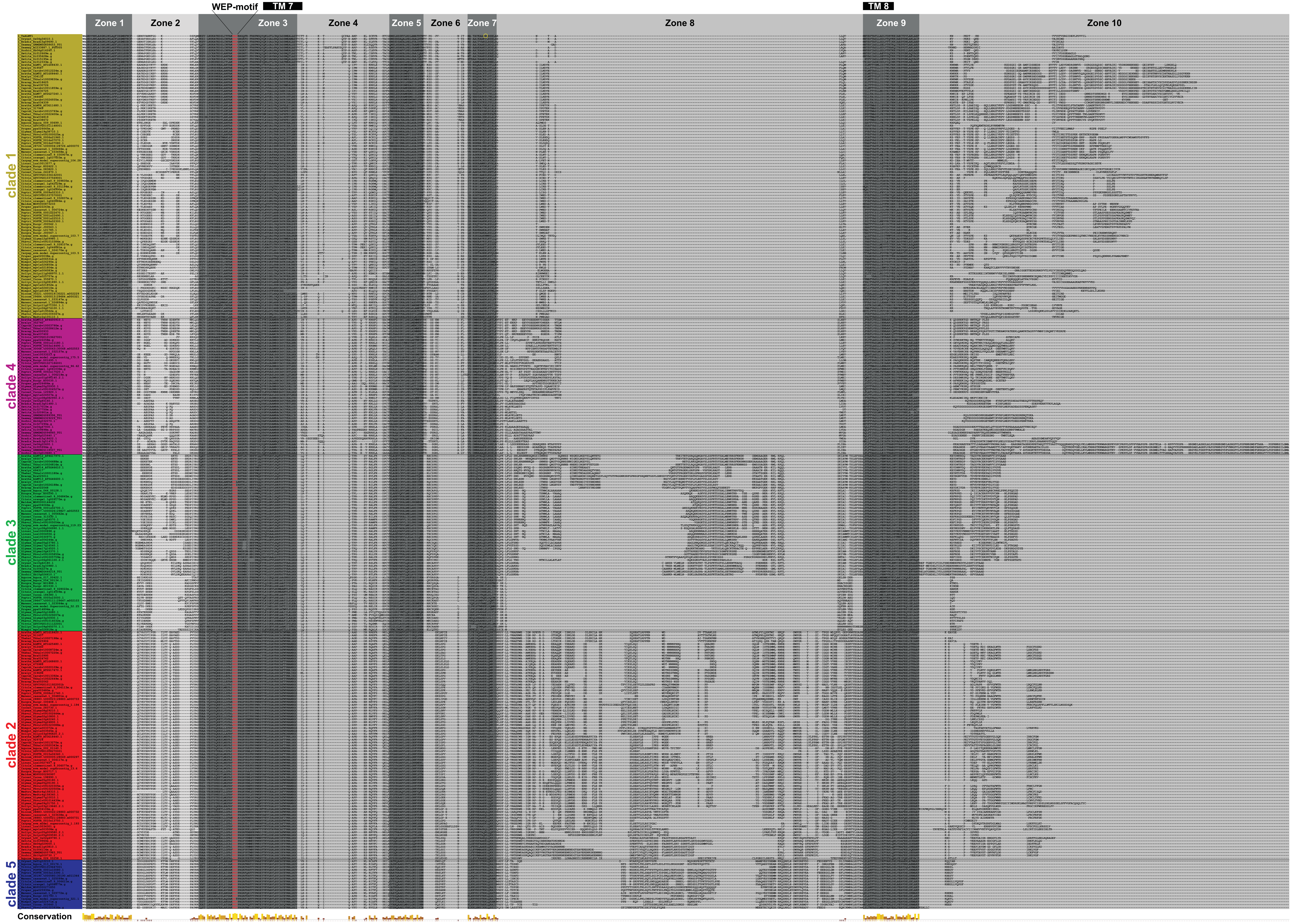
WEP-motif
TM 7
TM 8
Zone 1
Zone 2
Zone 3
Zone 4
Zone 5
Zone 6
Zone 7
Zone 8
Zone 9
Zone 10
clade 1
clade 4
clade 3
clade 2
clade 5
Conservation
